# Crystal structure and Hirshfeld surface analysis of 3-(cyclo­propyl­meth­oxy)-4-(di­fluoro­meth­oxy)-*N*-(pyridin-2-ylmeth­yl)benzamide

**DOI:** 10.1107/S2056989019012866

**Published:** 2019-09-20

**Authors:** G. Artheswari, V. Maheshwaran, N. Gautham

**Affiliations:** a CAS in Crystallography and Biophysics, University of Madras, Guindy Campus, Chennai 600 025, India

**Keywords:** crystal structure, benzamide, pyridine, cyclo­propane, supra­molecular features, hydrogen bonding, C—H⋯π inter­actions, Hirshfeld surface analysis

## Abstract

The title compound, C_18_H_18_F_2_N_2_O_3_, crystallizes with two independent mol­ecules (*A* and *B*) in the asymmetric unit. They differ essentially in the orientation of the pyridine ring with respect to the benzene ring; these two rings are inclined to each other by 53.3 (2)° in mol­ecule *A* and by 72.9 (2)° in mol­ecule *B*. The 3-(cyclo­propyl­meth­oxy) side chain has an extended conformation in both mol­ecules. The two mol­ecules are linked by a pair of C—H⋯O hydrogen bonds and two C—H⋯π inter­actions, forming an *A*–*B* unit. In the crystal, this unit is linked by N—H⋯O hydrogen bonds, forming a zigzag –*A*–*B*–*A*–*B*– chain along [001]. The chains are linked by C—H⋯N and C—H⋯F hydrogen bonds to form layers parallel to the *ac* plane. Finally, the layers are linked by a third C—H⋯π inter­action, forming a three-dimensional structure. The major contributions to the Hirshfeld surface are those due to H⋯H contacts (39.7%), followed by F⋯H/H⋯F contacts (19.2%).

## Abstract 2

The title *N*-(pyridin-2-ylmeth­yl)benzamide derivative, crystallizes with two independent mol­ecules (*A* and *B*) in the asymmetric unit, which differ essentially in the orientation of the pyridine ring with respect to the benzene ring, with the two rings being inclined to each other by 53.3 (2) and 72.9 (2)° in mol­ecules *A* and *B*, respectively.

## Chemical context   

Amides containing tri­fluoro­methyl substituents are important in both agrochemical research and pharmaceutical chemistry (Jeschke *et al.*, 2007 ▸; Jeschke, 2004 ▸; Leroux *et al.*, 2005 ▸). Amides show a broad spectrum of pharmacological properties, including anti­bacterial (Manojkumar *et al.* 2013*a* ▸), anti-inflammatory, anti­oxidant, analgesic and anti­viral activity (Manojkumar *et al.*, 2013*b* ▸). They also act as fungicides (Liu *et al.*, 2004*a* ▸), agaricides (Shiga *et al.*, 2003 ▸) and insecticides (Liu *et al.*, 2004*b* ▸). Following our inter­est in such compounds, we report herein on the synthesis, crystal structure and Hirshfeld surface analysis of the title compound, 3-(cyclo­propyl­meth­oxy)-4-(di­fluoro­meth­oxy)-*N*-(pyridin-2-ylmeth­yl)benzamide.
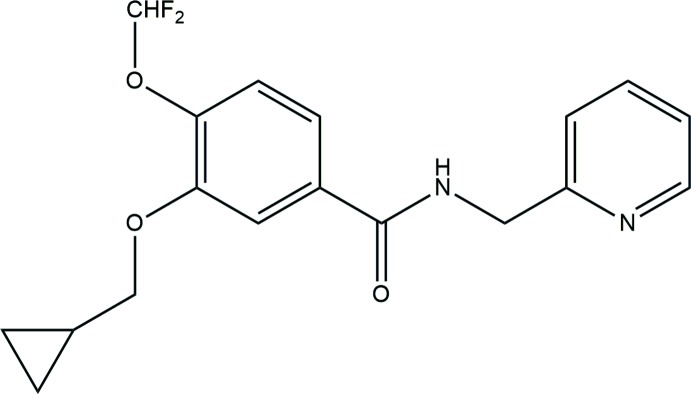


## Structural commentary   

**Figure 1:**
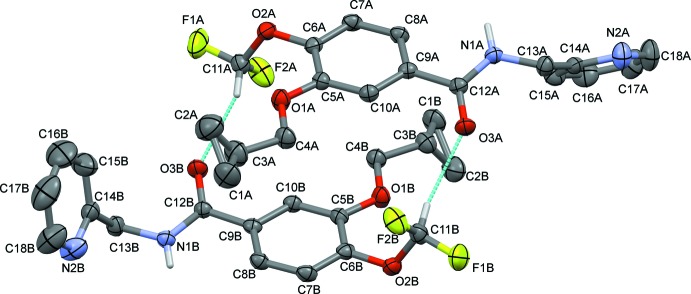
The mol­ecular structure of the title compound, showing the atomic labelling and the displacement ellipsoids drawn at 30% probability level. Hydrogen bonds (Table 1 ▸) are shown as dashed lines.

**Figure 2:**
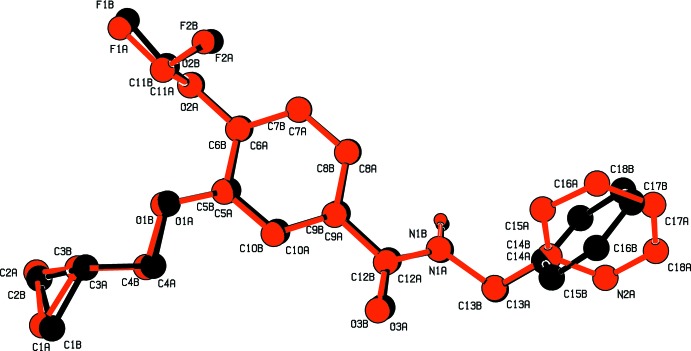
Structural overlay of inverted mol­ecule *B* (black) on mol­ecule *A* (red). Hydrogen atoms have been omitted for clarity.

The asymmetric unit of the title compound contains two crystallographically independent mol­ecules (*A* and *B*; Fig. 1 ▸). The overall conformation of the *A* and *B* mol­ecules differs in the orientation of the pyridine ring with respect to the benzene ring, as shown in the mol­ecular overlap figure [Fig. 2 ▸; inverted mol­ecule *B* (black) on mol­ecule *A* (red), with an r.m.s. deviation of 0.641 Å]. The dihedral angle between the benzamide ring and the pyridine ring is 53.3 (2)° in mol­ecule *A* and 72.9 (2)° in mol­ecule *B*. The cyclo­propane ring makes a dihedral angle of 57.7 (5)° with the benzene ring in mol­ecule *A* and 58.7 (4)° in mol­ecule *B*. The sum of the bond angles around atom N1 (359.9°) is in accordance with *sp* ^2^ hybridization in both mol­ecules. The bond lengths and bond angles in both mol­ecules are comparable with those reported for a similar compound, 3-(cyclo­propyl­meth­oxy)-*N*-(3,5-di­chloro­pyridin-4-yl)-4-(di­fluoro­meth­oxy) benzamide (Viertelhaus *et al.*, 2013 ▸), that crystallizes with three independent mol­ecules in the asymmetric unit.

## Supra­molecular features   

**Figure 3:**
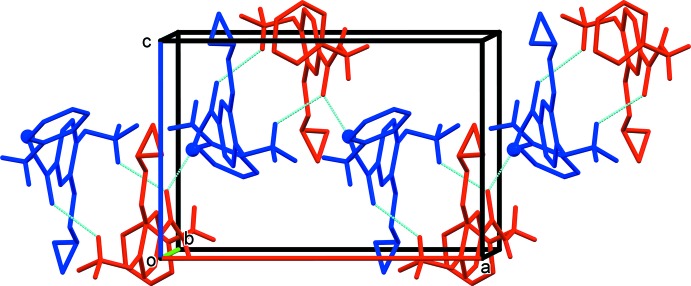
A partial view along the *b* axis of the crystal packing of the title compound (colour code: mol­ecule *A* blue, mol­ecule *B* red). The N—H⋯O and C—H⋯O hydrogen bonds (Table 1 ▸) are shown as dashed lines and, for clarity, only the H atoms involved in these inter­actions have been included.

**Figure 4:**
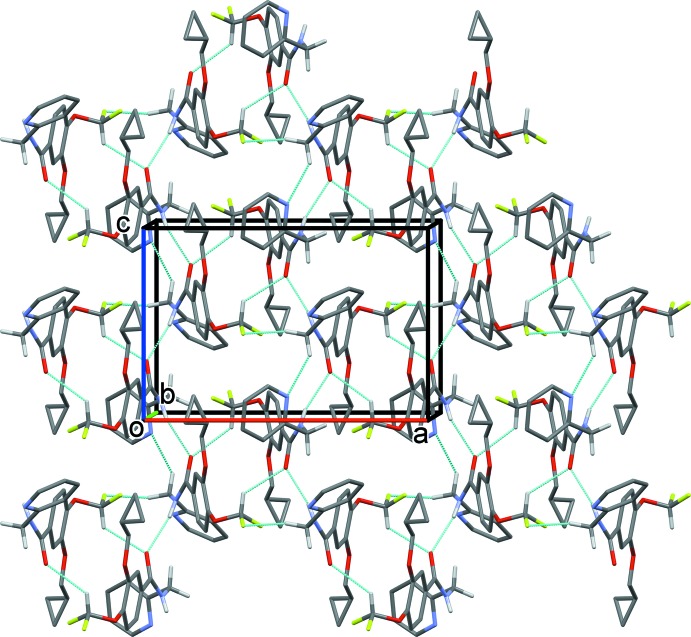
The crystal packing of the title compound, viewed along the *b* axis. The hydrogen bonds (Table 1 ▸) are shown as dashed lines. For clarity, only the H atoms involved in the inter­molecular inter­actions have been included.

**Figure 5:**
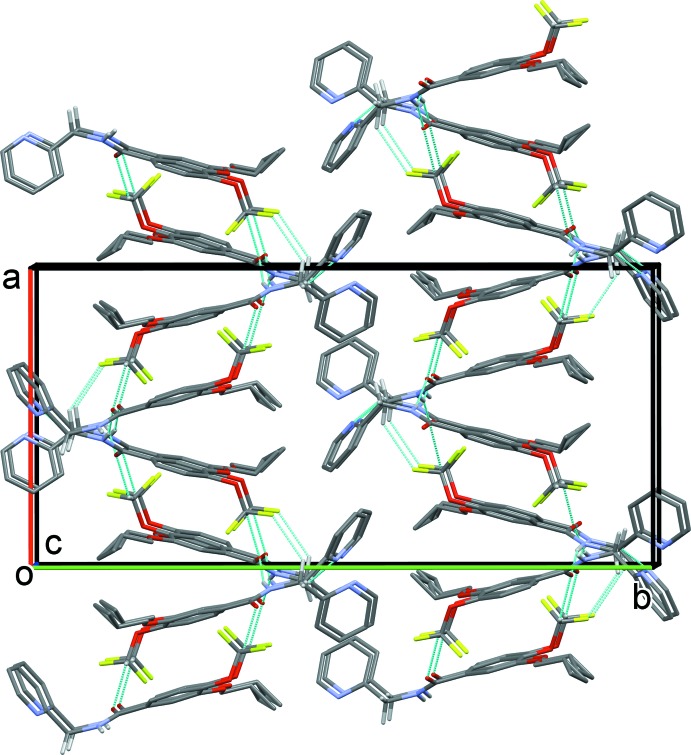
The crystal packing of the title compound, viewed along the *c* axis. The hydrogen bonds are shown as dashed lines (Table 1 ▸). For clarity, only the H atoms involved in the inter­molecular inter­actions have been included.

In the crystal, the *A* and *B* mol­ecules are linked by C—H⋯O and C—H⋯π inter­actions (Table 1 ▸ and Fig. 1 ▸), forming A-B units, which are in turn linked by N—H⋯O hydrogen bonds, forming chains propagating along the *c*-axis direction (Table 1 ▸ and Fig. 3 ▸). The chains are linked by C—H⋯O, C—H⋯N and C—H⋯F hydrogen bonds, forming layers lying parallel to the *ac* plane (Table 1 ▸ and Fig. 4 ▸). A third C—H⋯π inter­action links the layers to form a supra­molecular three-dimensional structure (Fig. 5 ▸).

## Database survey   

A search of the Cambridge Structural Database (CSD, Version 4.0, last update May 2019; Groom *et al.*, 2016 ▸) for the (cyclo­propyl­meth­oxy)benzene skeleton gave twelve hits for nine structures. Only three mol­ecules resemble the title compound. The most similar is 3-(cyclo­propyl­meth­oxy)-*N*-(3,5-di­chloro­pyridin-4-yl)-4-(di­fluoro­meth­oxy) benzamide (CSD refcode PEDWOM; Viertelhaus *et al.*, 2013 ▸). It is known as Roflumilast (trade names Daxas, Daliresp), a drug that has anti-inflammatory properties and is used in the treatment of chronic obstructive pulmonary disease (Hohlfeld *et al.*, 2008 ▸). The authors (Viertelhaus *et al.*, 2013 ▸) have made a variable temperature study of this compound (CSD entries PEDWOM at 100 K, PEDWOM01 at 343 K, PEDWOM02/PEDWOM03 at 298 K) in relation to a reversible single-crystal to single-crystal phase transition at 323 K. The compound crystallizes in the monoclinic *P*2_1_/*n* space group with three independent mol­ecules in the asymmetric unit. The high temperature phase at 343 K also crystallizes in space group *P*2_1_/*n* but with only one mol­ecule in the asymmetric unit, the length of the *b* axis being reduced by around a third. Here, the compound has a disordered di­fluoro­meth­oxy group. The overall conformations of the mol­ecules in all four entries (PEDWOM at 100 K, PEDWOM01 at 343 K, PEDWOM02/PEDWOM03 at 298 K) are very similar. Considering the low-temperature phase PEDWOM only, in each mol­ecule the benzene and pyridine rings are positioned almost perpendicular to each other, with dihedral angles of 88.38 (14), 89.34 (14) and 84.72 (14)°, compared to 53.3 (2) and 72.9 (2)° for mol­ecules *A* and *B*, respectively, in the title compound. In PEDWOM the cyclo­propane ring makes dihedral angles of 55.43 (3), 49.6 (3) and 50.9 (3)° with the corresponding benzene ring. These dihedral angles are very similar to those observed in the title structure [57.7 (5)° in mol­ecule *A* and 58.7 (4)° in mol­ecule *B*]. In the second compound, methyl 3-(cyclo­propyl­meth­oxy)-4-hy­droxy­benzoate (DUSXOF; Hou *et al.*, 2010 ▸), the cyclo­propane ring is inclined to the benzene ring by 63.34 (10)°, while in the third compound, methyl 3,4-bis­(cyclo­propyl­meth­oxy)benzo­ate (URAWEQ; Cheng *et al.*, 2011 ▸), this dihedral angle is smaller at 45.49 (11)°.

## Hirshfeld surface analysis   

**Figure 6:**
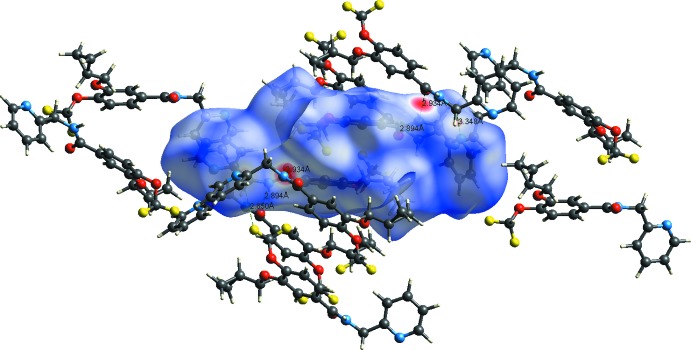
A view of the Hirshfeld surface of the title compound mapped over *d*_norm_, showing the various inter­molecular contacts in the crystal.

**Figure 7:**
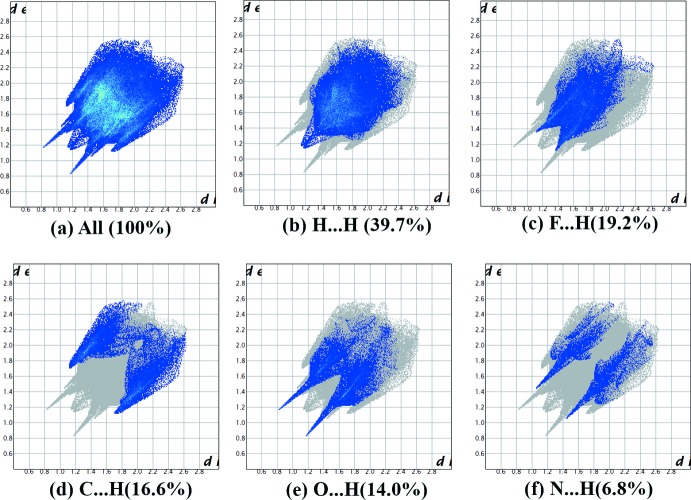
(*a*) The full two-dimensional fingerprint plot for the title compound, and fingerprint plots delineated into (*b*) H⋯H, (*c*) F⋯H/H⋯F, (*d*) C⋯H/H⋯C, (*e*) O⋯H/H⋯O and (*f*) N⋯H/H⋯N contacts.

The Hirshfeld surface analysis (Spackman & Jayatilaka, 2009 ▸) and the associated two-dimensional fingerprint plots (McKinnon *et al.*, 2007 ▸) were performed with *CrystalExplorer17* (Turner *et al.*, 2017 ▸). The Hirshfeld surface mapped over *d*_norm_ in the colour range of −0.4869 to 1.4157 arbitrary units, and the inter­molecular contacts are illustrated in Fig. 6 ▸. The red spots on the surface indicate the inter­molecular contacts involved in hydrogen bonding (Table 1 ▸). The two-dimensional fingerprint plots are given in Fig. 7 ▸. They reveal that the principal inter­molecular contacts are H⋯H at 39.7% (Fig. 7 ▸ *b*), followed by F⋯H/ H⋯F at 19.2% (Fig. 7 ▸ *c*), C⋯H/H⋯C at 16.6% (Fig. 7 ▸ *d*), O⋯H/ H⋯O at 14.0% (Fig. 7 ▸ *e*), N⋯H/H⋯N at 6.8% (Fig. 7 ▸ *f*). Hence, the H⋯H and F⋯H/H⋯F inter­molecular contacts are the most abundant in the crystal packing, and make the most significant contributions to the total Hirshfeld surfaces.

## Synthesis and crystallization   

A mixture of 4-(di­fluoro­meth­yl)-3-hy­droxy­benzoic acid (2 mmol), (chloro­meth­yl)cyclo­propane (2 mmol) and 2-picolyl­amine (3 mmol) with PPh_3_ (0.2 mmol) in methanol were heated first to 393 K for 2 h in the presence of the inexpensive ionic liquid tetra­butyl­ammonium bromide (TBAB). The reaction was monitored by TLC, and on completion the reaction mixture was allowed to cool to room temperature, then filtered to remove the insoluble solids. The filtered solid was then washed with di­chloro­methane. Excess solvents were removed under reduced pressure and the obtained crude product was purified by crystallization using 1:1 ratio of chloro­form and methanol. Colourless block-like crystals were obtained after two days.

## Refinement   

Crystal data, data collection and structure refinement details are summarized in Table 2 ▸. The NH H atoms were located in difference-Fourier maps and refined freely. The C-bound H atoms were positioned geometrically (C—H = 0.93—0.98 Å) and allowed to ride on their parent atoms, with *U*_iso_(H) =1.5*U*_eq_(C-meth­yl) and 1.2*U*_eq_(C) for other H atoms. The absolute structure of the mol­ecules in the crystal are unknown; the Flack parameter refined to 0.6 (3).

## Supplementary Material

Crystal structure: contains datablock(s) global, I. DOI: 10.1107/S2056989019012866/su5514sup1.cif

Structure factors: contains datablock(s) I. DOI: 10.1107/S2056989019012866/su5514Isup2.hkl

Supporting information file. DOI: 10.1107/S2056989019012866/su5514Isup3.cml

CCDC reference: 1954107

Additional supporting information:  crystallographic information; 3D view; checkCIF report

## Figures and Tables

**Table 1:** Hydrogen-bond geometry (Å, °) *Cg*2, *Cg*3 and *Cg*6 are the centroids of the N2*A*/C14*A*–C18*A*, C5*A*–C10*A* and C5*B*–C10*B* rings, respectively.

*D*—H⋯*A*	*D*—H	H⋯*A*	*D*⋯*A*	*D*—H⋯*A*
C11*A*—H11*A*⋯O3*B*	0.98	2.44	3.136 (5)	128
C11*B*—H11*B*⋯O3*A*	0.98	2.47	3.210 (5)	132
C4*B*—H4*B*1⋯*Cg*3	0.97	2.87	3.689 (4)	143
C4*A*—H4*A*2⋯*Cg*6	0.97	2.90	3.717 (5)	143
N1*A*—H1*A*⋯O3*B* ^i^	0.84 (4)	2.08 (4)	2.895 (4)	163 (4)
N1*B*—H1*B*⋯O3*A* ^ii^	0.75 (4)	2.21 (4)	2.939 (4)	164 (4)
C13*A*—H13*C*⋯N2*B* ^iii^	0.97	2.55	3.347 (5)	140
C13*A*—H13*D*⋯F1*A* ^i^	0.97	2.52	3.294 (6)	136
C13*B*—H13*B*⋯*Cg*2^iv^	0.97	2.73	3.748 (4)	137

Symmetry codes: (i) 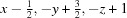; (ii) 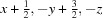; (iii) 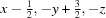; (iv) 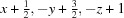.

**Table 2:** Experimental details

Crystal data	
Chemical formula	C_18_H_18_F_2_N_2_O_3_
*M*_r_	348.34
Crystal system, space group	Orthorhombic, *P*2_1_2_1_2_1_
Temperature (K)	293
*a*, *b*, *c* (Å)	13.4775 (12), 28.026 (3), 9.1085 (9)
*V* (Å^3^)	3440.5 (6)
*Z*	8
Radiation type	Mo *K*α
μ (mm^−1^)	0.11
Crystal size (mm)	0.30 × 0.25 × 0.20

Data collection	
Diffractometer	Bruker SMART APEXII area-detector diffractometer
Absorption correction	Multi-scan (*SADABS*; Bruker, 2008 ▸)
*T*_min_, *T*_max_	0.642, 0.785
No. of measured, independent and observed [*I* > 2σ(*I*)] reflections	33263, 8521, 5868
*R*_int_	0.037
(sin θ/λ)_max_ (Å^−1^)	0.668

Refinement	
*R*[*F* ^2^ > 2σ(*F* ^2^)], *wR*(*F* ^2^), *S*	0.053, 0.167, 1.02
No. of reflections	8521
No. of parameters	459
Δρ_max_, Δρ_min_ (e Å^−3^)	0.67, −0.24

Computer programs: *APEX2* and *SAINT* (Bruker, 2008 ▸), *SHELXS2013/1* (Sheldrick, 2008 ▸), *ORTEP-3 for Windows* (Farrugia, 2012 ▸) and *Mercury* (Macrae *et al.*, 2008 ▸), *SHELXL2018/3* (Sheldrick, 2015 ▸) and *PLATON* (Spek, 2009 ▸).

## supplementary crystallographic information

### Crystal data

**Table:**

C_18_H_18_F_2_N_2_O_3_	*D*_x_ = 1.345 Mg m^−^^3^
*M**_r_* = 348.34	Mo *K*α radiation, λ = 0.71073 Å
Orthorhombic, *P*2_1_2_1_2_1_	Cell parameters from 8521 reflections
*a* = 13.4775 (12) Å	θ = 1.5–28.4°
*b* = 28.026 (3) Å	µ = 0.11 mm^−^^1^
*c* = 9.1085 (9) Å	*T* = 293 K
*V* = 3440.5 (6) Å^3^	BLOCK, colourless
*Z* = 8	0.30 × 0.25 × 0.20 mm
*F*(000) = 1456	

### Data collection

**Table:**

Bruker SMART APEXII area-detector diffractometer	5868 reflections with *I* > 2σ(*I*)
ω and φ scans	*R*_int_ = 0.037
Absorption correction: multi-scan (*SADABS*; Bruker, 2008)	θ_max_ = 28.4°, θ_min_ = 1.5°
*T*_min_ = 0.642, *T*_max_ = 0.785	*h* = −16→17
33263 measured reflections	*k* = −37→37
8521 independent reflections	*l* = −10→12

### Refinement

**Table:**

Refinement on *F*^2^	Primary atom site location: structure-invariant direct methods
Least-squares matrix: full	Secondary atom site location: difference Fourier map
*R*[*F*^2^ > 2σ(*F*^2^)] = 0.053	Hydrogen site location: mixed
*wR*(*F*^2^) = 0.167	H atoms treated by a mixture of independent and constrained refinement
*S* = 1.02	*w* = 1/[σ^2^(*F*_o_^2^) + (0.0901*P*)^2^ + 0.6343*P*] where *P* = (*F*_o_^2^ + 2*F*_c_^2^)/3
8521 reflections	(Δ/σ)_max_ < 0.001
459 parameters	Δρ_max_ = 0.67 e Å^−^^3^
0 restraints	Δρ_min_ = −0.24 e Å^−^^3^

### Special details

**Table:**

Geometry. All esds (except the esd in the dihedral angle between two l.s. planes) are estimated using the full covariance matrix. The cell esds are taken into account individually in the estimation of esds in distances, angles and torsion angles; correlations between esds in cell parameters are only used when they are defined by crystal symmetry. An approximate (isotropic) treatment of cell esds is used for estimating esds involving l.s. planes.

### Fractional atomic coordinates and isotropic or equivalent isotropic displacement parameters (Å^2^)

**Table:**

	*x*	*y*	*z*	*U*_iso_*/*U*_eq_	
C3A	0.6926 (5)	0.85621 (17)	0.0399 (6)	0.0849 (15)	
H3A	0.760513	0.868025	0.052106	0.102*	
C3B	0.8930 (3)	0.64609 (14)	0.4443 (5)	0.0594 (10)	
H3B	0.824697	0.634683	0.432638	0.071*	
C1B	0.9416 (4)	0.63426 (16)	0.5869 (5)	0.0770 (14)	
H1B1	0.988434	0.657112	0.626991	0.092*	
H1B2	0.902904	0.617075	0.659464	0.092*	
C1A	0.6470 (6)	0.8684 (2)	−0.0992 (6)	0.0997 (19)	
H1A1	0.686226	0.886135	−0.169794	0.120*	
H1A2	0.600245	0.845892	−0.141389	0.120*	
C2A	0.6200 (6)	0.8918 (2)	0.0358 (7)	0.111 (2)	
H2A1	0.641359	0.924536	0.049262	0.134*	
H2A2	0.555580	0.884387	0.077600	0.134*	
C2B	0.9698 (4)	0.60887 (18)	0.4527 (6)	0.0821 (14)	
H2B1	0.948720	0.575924	0.442855	0.098*	
H2B2	1.034168	0.615922	0.410413	0.098*	
C4B	0.9158 (3)	0.69265 (12)	0.3746 (4)	0.0533 (9)	
H4B1	0.871794	0.717173	0.412054	0.064*	
H4B2	0.983671	0.701907	0.395770	0.064*	
C4A	0.6728 (4)	0.80972 (14)	0.1111 (4)	0.0599 (10)	
H4A1	0.605344	0.799607	0.090866	0.072*	
H4A2	0.717818	0.785685	0.073220	0.072*	
C5A	0.6758 (3)	0.77698 (11)	0.3535 (4)	0.0421 (7)	
C5B	0.9153 (3)	0.72633 (11)	0.1320 (4)	0.0418 (7)	
C10A	0.6486 (3)	0.73121 (12)	0.3097 (4)	0.0423 (7)	
H10A	0.636984	0.725073	0.210853	0.051*	
C10B	0.9444 (3)	0.77079 (12)	0.1793 (4)	0.0425 (7)	
H10B	0.956199	0.775829	0.278668	0.051*	
C9A	0.6385 (2)	0.69462 (11)	0.4113 (3)	0.0398 (7)	
C9B	0.9564 (2)	0.80857 (11)	0.0804 (3)	0.0393 (7)	
C8B	0.9374 (3)	0.80117 (12)	−0.0676 (4)	0.0443 (7)	
H8B	0.944975	0.825994	−0.134414	0.053*	
C8A	0.6566 (3)	0.70345 (12)	0.5597 (4)	0.0450 (8)	
H8A	0.649867	0.679050	0.628203	0.054*	
C7A	0.6847 (3)	0.74862 (12)	0.6046 (4)	0.0467 (8)	
H7A	0.696901	0.754677	0.703354	0.056*	
C7B	0.9069 (3)	0.75644 (12)	−0.1152 (4)	0.0471 (8)	
H7B	0.894040	0.751332	−0.214280	0.057*	
C6A	0.6947 (3)	0.78472 (11)	0.5024 (4)	0.0428 (7)	
C6B	0.8957 (3)	0.71980 (11)	−0.0171 (4)	0.0441 (8)	
C11B	0.7764 (3)	0.65960 (13)	−0.0361 (4)	0.0556 (9)	
H11B	0.770549	0.653960	0.069771	0.067*	
C11A	0.8125 (3)	0.84507 (12)	0.5158 (4)	0.0523 (9)	
H11A	0.820805	0.846015	0.408994	0.063*	
C12A	0.6111 (3)	0.64577 (11)	0.3570 (3)	0.0399 (7)	
C12B	0.9851 (3)	0.85610 (11)	0.1403 (3)	0.0394 (7)	
C13B	1.0486 (3)	0.93630 (12)	0.0893 (4)	0.0527 (9)	
H13A	1.112422	0.944908	0.047812	0.063*	
H13B	1.053716	0.938497	0.195349	0.063*	
C13A	0.5474 (3)	0.56661 (11)	0.4218 (4)	0.0504 (9)	
H13C	0.558990	0.560555	0.318366	0.060*	
H13D	0.477237	0.561821	0.440582	0.060*	
C14A	0.6056 (3)	0.53127 (11)	0.5106 (4)	0.0468 (8)	
C14B	0.9714 (3)	0.97029 (12)	0.0361 (5)	0.0574 (10)	
C15A	0.6971 (3)	0.54131 (14)	0.5656 (5)	0.0604 (10)	
H15A	0.726593	0.570913	0.550449	0.072*	
C15B	0.8969 (5)	0.9869 (2)	0.1237 (8)	0.0966 (18)	
H15B	0.893675	0.977774	0.221716	0.116*	
C16A	0.7455 (4)	0.50557 (19)	0.6458 (7)	0.0808 (14)	
H16A	0.808325	0.511136	0.684333	0.097*	
C16B	0.8265 (6)	1.0173 (3)	0.0664 (11)	0.128 (3)	
H16B	0.774980	1.029011	0.124056	0.154*	
C17A	0.7010 (5)	0.46329 (18)	0.6668 (7)	0.0911 (17)	
H17A	0.732284	0.439371	0.720385	0.109*	
C17B	0.8351 (6)	1.0297 (2)	−0.0794 (11)	0.122 (3)	
H17B	0.787592	1.048970	−0.124001	0.146*	
C18A	0.6109 (5)	0.45618 (16)	0.6094 (7)	0.0910 (17)	
H18A	0.580764	0.426749	0.624739	0.109*	
C18B	0.9148 (6)	1.0132 (2)	−0.1574 (7)	0.108 (2)	
H18B	0.922244	1.023375	−0.253996	0.130*	
N1A	0.5726 (3)	0.61610 (10)	0.4539 (3)	0.0465 (7)	
N1B	1.0244 (3)	0.88763 (10)	0.0482 (4)	0.0473 (7)	
N2B	0.9813 (4)	0.98388 (15)	−0.1035 (4)	0.0834 (13)	
N2A	0.5614 (3)	0.48899 (12)	0.5308 (5)	0.0714 (10)	
O1B	0.9022 (2)	0.68714 (8)	0.2187 (3)	0.0538 (6)	
O1A	0.6868 (2)	0.81526 (8)	0.2653 (3)	0.0573 (7)	
O2A	0.7199 (2)	0.83023 (8)	0.5524 (3)	0.0547 (7)	
O2B	0.8688 (2)	0.67504 (9)	−0.0719 (3)	0.0547 (7)	
O3A	0.6272 (2)	0.63485 (8)	0.2278 (3)	0.0527 (6)	
O3B	0.9710 (2)	0.86563 (9)	0.2718 (3)	0.0555 (7)	
F2B	0.7076 (2)	0.69107 (10)	−0.0828 (3)	0.0810 (8)	
F1A	0.8220 (3)	0.88794 (10)	0.5731 (5)	0.1111 (12)	
F2A	0.8810 (2)	0.81731 (11)	0.5780 (4)	0.0885 (9)	
F1B	0.7623 (3)	0.61959 (10)	−0.1115 (4)	0.0974 (10)	
H1A	0.552 (3)	0.6261 (14)	0.535 (5)	0.053 (11)*	
H1B	1.040 (3)	0.8804 (13)	−0.028 (4)	0.039 (10)*	

### Atomic displacement parameters (Å^2^)

**Table:**

	*U*^11^	*U*^22^	*U*^33^	*U*^12^	*U*^13^	*U*^23^
C3A	0.108 (4)	0.075 (3)	0.072 (3)	0.002 (3)	−0.004 (3)	0.018 (2)
C3B	0.059 (2)	0.059 (2)	0.060 (2)	−0.0020 (18)	0.0005 (19)	0.0089 (18)
C1B	0.107 (4)	0.073 (3)	0.051 (3)	−0.007 (2)	−0.003 (2)	0.016 (2)
C1A	0.146 (6)	0.095 (4)	0.059 (3)	0.009 (4)	−0.003 (3)	0.021 (3)
C2A	0.140 (6)	0.097 (4)	0.097 (5)	0.029 (4)	0.011 (4)	0.001 (3)
C2B	0.088 (4)	0.071 (3)	0.087 (4)	0.017 (2)	0.003 (3)	0.019 (3)
C4B	0.064 (2)	0.0516 (18)	0.044 (2)	−0.0016 (17)	−0.0037 (17)	0.0031 (16)
C4A	0.074 (3)	0.058 (2)	0.047 (2)	−0.0015 (19)	−0.0061 (19)	0.0088 (17)
C5A	0.0406 (19)	0.0444 (16)	0.0414 (18)	0.0031 (13)	0.0003 (14)	0.0035 (13)
C5B	0.0406 (18)	0.0454 (15)	0.0395 (18)	0.0010 (13)	0.0013 (13)	0.0041 (13)
C10A	0.0437 (19)	0.0486 (16)	0.0346 (17)	0.0018 (14)	−0.0049 (13)	0.0002 (13)
C10B	0.045 (2)	0.0489 (16)	0.0334 (17)	0.0004 (14)	−0.0027 (13)	0.0028 (13)
C9A	0.0374 (17)	0.0476 (15)	0.0344 (16)	0.0033 (13)	−0.0024 (13)	−0.0009 (13)
C9B	0.0331 (16)	0.0482 (16)	0.0365 (17)	0.0019 (12)	0.0008 (12)	0.0013 (13)
C8B	0.0449 (18)	0.0546 (17)	0.0334 (17)	−0.0044 (14)	−0.0005 (13)	0.0037 (14)
C8A	0.0497 (19)	0.0513 (17)	0.0341 (18)	−0.0008 (14)	0.0002 (14)	0.0031 (14)
C7A	0.049 (2)	0.0578 (19)	0.0330 (17)	−0.0058 (16)	−0.0019 (14)	−0.0040 (15)
C7B	0.047 (2)	0.0619 (19)	0.0323 (17)	−0.0067 (16)	−0.0003 (14)	−0.0025 (15)
C6A	0.0392 (19)	0.0469 (16)	0.0425 (19)	−0.0003 (13)	0.0027 (14)	−0.0071 (14)
C6B	0.0366 (18)	0.0520 (17)	0.0437 (19)	−0.0042 (14)	0.0038 (14)	−0.0079 (14)
C11B	0.062 (3)	0.060 (2)	0.045 (2)	−0.0165 (18)	−0.0007 (17)	0.0005 (17)
C11A	0.052 (2)	0.0510 (18)	0.054 (2)	−0.0069 (16)	0.0001 (17)	0.0023 (16)
C12A	0.0417 (18)	0.0445 (15)	0.0337 (16)	0.0048 (13)	−0.0034 (13)	0.0018 (13)
C12B	0.0420 (18)	0.0430 (15)	0.0330 (16)	0.0010 (13)	−0.0020 (13)	0.0020 (13)
C13B	0.068 (3)	0.0457 (17)	0.044 (2)	−0.0066 (16)	−0.0037 (18)	0.0005 (15)
C13A	0.062 (2)	0.0428 (16)	0.047 (2)	−0.0025 (15)	−0.0055 (17)	−0.0020 (14)
C14A	0.056 (2)	0.0431 (16)	0.0416 (19)	0.0038 (15)	0.0052 (15)	−0.0040 (14)
C14B	0.071 (3)	0.0446 (17)	0.057 (2)	−0.0013 (17)	0.001 (2)	−0.0065 (16)
C15A	0.052 (2)	0.065 (2)	0.065 (3)	0.0005 (18)	−0.0015 (19)	0.0017 (19)
C15B	0.097 (4)	0.093 (3)	0.100 (4)	0.018 (3)	0.032 (4)	0.019 (3)
C16A	0.060 (3)	0.092 (3)	0.090 (4)	0.016 (2)	−0.011 (3)	0.004 (3)
C16B	0.105 (5)	0.116 (5)	0.163 (8)	0.044 (4)	0.028 (5)	0.004 (5)
C17A	0.116 (5)	0.067 (3)	0.091 (4)	0.027 (3)	−0.020 (3)	0.013 (3)
C17B	0.129 (6)	0.094 (4)	0.142 (7)	0.048 (4)	−0.048 (5)	−0.014 (4)
C18A	0.121 (5)	0.053 (2)	0.099 (4)	−0.005 (3)	−0.022 (4)	0.018 (2)
C18B	0.153 (7)	0.098 (4)	0.073 (4)	0.053 (4)	−0.029 (4)	−0.003 (3)
N1A	0.0626 (19)	0.0413 (13)	0.0355 (16)	−0.0002 (13)	0.0056 (13)	−0.0034 (12)
N1B	0.063 (2)	0.0443 (14)	0.0343 (16)	0.0007 (13)	0.0049 (14)	0.0010 (13)
N2B	0.115 (4)	0.083 (2)	0.052 (2)	0.035 (2)	−0.007 (2)	0.002 (2)
N2A	0.086 (3)	0.0524 (17)	0.076 (3)	−0.0080 (17)	−0.015 (2)	0.0087 (17)
O1B	0.0715 (19)	0.0454 (12)	0.0445 (14)	−0.0040 (12)	−0.0072 (12)	0.0056 (10)
O1A	0.075 (2)	0.0483 (13)	0.0487 (15)	−0.0047 (12)	−0.0070 (13)	0.0062 (11)
O2A	0.0512 (16)	0.0537 (13)	0.0593 (16)	−0.0064 (11)	0.0102 (12)	−0.0142 (12)
O2B	0.0600 (17)	0.0553 (13)	0.0488 (15)	−0.0113 (11)	0.0107 (12)	−0.0133 (11)
O3A	0.0662 (17)	0.0570 (13)	0.0349 (13)	−0.0037 (12)	0.0031 (11)	−0.0057 (10)
O3B	0.0734 (19)	0.0597 (14)	0.0336 (13)	−0.0128 (13)	0.0001 (12)	−0.0025 (11)
F2B	0.0607 (16)	0.0891 (17)	0.093 (2)	−0.0114 (13)	−0.0134 (14)	0.0211 (15)
F1A	0.095 (2)	0.0660 (15)	0.172 (4)	−0.0286 (15)	0.019 (2)	−0.0379 (19)
F2A	0.0591 (16)	0.1027 (19)	0.104 (2)	−0.0103 (14)	−0.0187 (15)	0.0355 (17)
F1B	0.110 (2)	0.0744 (16)	0.108 (2)	−0.0407 (16)	0.0166 (19)	−0.0294 (16)

### Geometric parameters (Å, º)

**Table:**

C3A—C2A	1.397 (9)	C7B—H7B	0.9300
C3A—C1A	1.449 (8)	C6A—O2A	1.396 (4)
C3A—C4A	1.479 (6)	C6B—O2B	1.398 (4)
C3A—H3A	0.9800	C11B—F1B	1.329 (4)
C3B—C2B	1.471 (6)	C11B—F2B	1.348 (5)
C3B—C4B	1.484 (5)	C11B—O2B	1.359 (5)
C3B—C1B	1.492 (6)	C11B—H11B	0.9800
C3B—H3B	0.9800	C11A—F1A	1.316 (4)
C1B—C2B	1.465 (7)	C11A—F2A	1.334 (5)
C1B—H1B1	0.9700	C11A—O2A	1.357 (5)
C1B—H1B2	0.9700	C11A—H11A	0.9800
C1A—C2A	1.441 (8)	C12A—O3A	1.235 (4)
C1A—H1A1	0.9700	C12A—N1A	1.319 (4)
C1A—H1A2	0.9700	C12B—O3B	1.242 (4)
C2A—H2A1	0.9700	C12B—N1B	1.328 (4)
C2A—H2A2	0.9700	C13B—N1B	1.452 (4)
C2B—H2B1	0.9700	C13B—C14B	1.492 (6)
C2B—H2B2	0.9700	C13B—H13A	0.9700
C4B—O1B	1.440 (4)	C13B—H13B	0.9700
C4B—H4B1	0.9700	C13A—N1A	1.458 (4)
C4B—H4B2	0.9700	C13A—C14A	1.500 (5)
C4A—O1A	1.426 (5)	C13A—H13C	0.9700
C4A—H4A1	0.9700	C13A—H13D	0.9700
C4A—H4A2	0.9700	C14A—N2A	1.339 (5)
C5A—O1A	1.349 (4)	C14A—C15A	1.361 (6)
C5A—C10A	1.392 (5)	C14B—N2B	1.333 (6)
C5A—C6A	1.397 (5)	C14B—C15B	1.365 (7)
C5B—O1B	1.365 (4)	C15A—C16A	1.400 (6)
C5B—C10B	1.375 (5)	C15A—H15A	0.9300
C5B—C6B	1.395 (5)	C15B—C16B	1.378 (10)
C10A—C9A	1.388 (5)	C15B—H15B	0.9300
C10A—H10A	0.9300	C16A—C17A	1.342 (8)
C10B—C9B	1.399 (4)	C16A—H16A	0.9300
C10B—H10B	0.9300	C16B—C17B	1.378 (11)
C9A—C8A	1.396 (5)	C16B—H16B	0.9300
C9A—C12A	1.501 (4)	C17A—C18A	1.337 (9)
C9B—C8B	1.388 (5)	C17A—H17A	0.9300
C9B—C12B	1.490 (4)	C17B—C18B	1.369 (11)
C8B—C7B	1.389 (5)	C17B—H17B	0.9300
C8B—H8B	0.9300	C18A—N2A	1.343 (6)
C8A—C7A	1.383 (5)	C18A—H18A	0.9300
C8A—H8A	0.9300	C18B—N2B	1.312 (7)
C7A—C6A	1.381 (5)	C18B—H18B	0.9300
C7A—H7A	0.9300	N1A—H1A	0.83 (4)
C7B—C6B	1.370 (5)	N1B—H1B	0.75 (4)
			
C2A—C3A—C1A	60.8 (4)	C7A—C6A—O2A	118.2 (3)
C2A—C3A—C4A	120.9 (6)	C7A—C6A—C5A	121.5 (3)
C1A—C3A—C4A	120.9 (5)	O2A—C6A—C5A	120.2 (3)
C2A—C3A—H3A	114.6	C7B—C6B—C5B	121.1 (3)
C1A—C3A—H3A	114.6	C7B—C6B—O2B	117.9 (3)
C4A—C3A—H3A	114.6	C5B—C6B—O2B	121.0 (3)
C2B—C3B—C4B	120.0 (4)	F1B—C11B—F2B	106.9 (3)
C2B—C3B—C1B	59.2 (3)	F1B—C11B—O2B	106.0 (3)
C4B—C3B—C1B	118.5 (4)	F2B—C11B—O2B	110.3 (3)
C2B—C3B—H3B	115.8	F1B—C11B—H11B	111.2
C4B—C3B—H3B	115.8	F2B—C11B—H11B	111.2
C1B—C3B—H3B	115.8	O2B—C11B—H11B	111.2
C2B—C1B—C3B	59.7 (3)	F1A—C11A—F2A	107.2 (4)
C2B—C1B—H1B1	117.8	F1A—C11A—O2A	105.8 (3)
C3B—C1B—H1B1	117.8	F2A—C11A—O2A	110.7 (3)
C2B—C1B—H1B2	117.8	F1A—C11A—H11A	111.0
C3B—C1B—H1B2	117.8	F2A—C11A—H11A	111.0
H1B1—C1B—H1B2	114.9	O2A—C11A—H11A	111.0
C2A—C1A—C3A	57.8 (4)	O3A—C12A—N1A	123.4 (3)
C2A—C1A—H1A1	118.0	O3A—C12A—C9A	119.8 (3)
C3A—C1A—H1A1	118.0	N1A—C12A—C9A	116.8 (3)
C2A—C1A—H1A2	118.0	O3B—C12B—N1B	121.8 (3)
C3A—C1A—H1A2	118.0	O3B—C12B—C9B	120.3 (3)
H1A1—C1A—H1A2	115.2	N1B—C12B—C9B	117.8 (3)
C3A—C2A—C1A	61.3 (4)	N1B—C13B—C14B	111.1 (3)
C3A—C2A—H2A1	117.6	N1B—C13B—H13A	109.4
C1A—C2A—H2A1	117.6	C14B—C13B—H13A	109.4
C3A—C2A—H2A2	117.6	N1B—C13B—H13B	109.4
C1A—C2A—H2A2	117.6	C14B—C13B—H13B	109.4
H2A1—C2A—H2A2	114.7	H13A—C13B—H13B	108.0
C1B—C2B—C3B	61.1 (3)	N1A—C13A—C14A	113.4 (3)
C1B—C2B—H2B1	117.7	N1A—C13A—H13C	108.9
C3B—C2B—H2B1	117.7	C14A—C13A—H13C	108.9
C1B—C2B—H2B2	117.7	N1A—C13A—H13D	108.9
C3B—C2B—H2B2	117.7	C14A—C13A—H13D	108.9
H2B1—C2B—H2B2	114.8	H13C—C13A—H13D	107.7
O1B—C4B—C3B	107.5 (3)	N2A—C14A—C15A	122.4 (4)
O1B—C4B—H4B1	110.2	N2A—C14A—C13A	115.2 (4)
C3B—C4B—H4B1	110.2	C15A—C14A—C13A	122.4 (3)
O1B—C4B—H4B2	110.2	N2B—C14B—C15B	122.2 (5)
C3B—C4B—H4B2	110.2	N2B—C14B—C13B	115.0 (4)
H4B1—C4B—H4B2	108.5	C15B—C14B—C13B	122.8 (4)
O1A—C4A—C3A	108.2 (4)	C14A—C15A—C16A	117.8 (4)
O1A—C4A—H4A1	110.1	C14A—C15A—H15A	121.1
C3A—C4A—H4A1	110.1	C16A—C15A—H15A	121.1
O1A—C4A—H4A2	110.1	C14B—C15B—C16B	119.8 (6)
C3A—C4A—H4A2	110.1	C14B—C15B—H15B	120.1
H4A1—C4A—H4A2	108.4	C16B—C15B—H15B	120.1
O1A—C5A—C10A	126.2 (3)	C17A—C16A—C15A	119.9 (5)
O1A—C5A—C6A	115.8 (3)	C17A—C16A—H16A	120.1
C10A—C5A—C6A	118.0 (3)	C15A—C16A—H16A	120.1
O1B—C5B—C10B	125.8 (3)	C17B—C16B—C15B	117.6 (7)
O1B—C5B—C6B	115.7 (3)	C17B—C16B—H16B	121.2
C10B—C5B—C6B	118.5 (3)	C15B—C16B—H16B	121.2
C9A—C10A—C5A	121.0 (3)	C18A—C17A—C16A	118.8 (5)
C9A—C10A—H10A	119.5	C18A—C17A—H17A	120.6
C5A—C10A—H10A	119.5	C16A—C17A—H17A	120.6
C5B—C10B—C9B	121.1 (3)	C18B—C17B—C16B	118.7 (6)
C5B—C10B—H10B	119.4	C18B—C17B—H17B	120.6
C9B—C10B—H10B	119.4	C16B—C17B—H17B	120.6
C10A—C9A—C8A	119.8 (3)	C17A—C18A—N2A	123.9 (5)
C10A—C9A—C12A	118.6 (3)	C17A—C18A—H18A	118.0
C8A—C9A—C12A	121.6 (3)	N2A—C18A—H18A	118.0
C8B—C9B—C10B	119.4 (3)	N2B—C18B—C17B	123.6 (6)
C8B—C9B—C12B	122.5 (3)	N2B—C18B—H18B	118.2
C10B—C9B—C12B	118.1 (3)	C17B—C18B—H18B	118.2
C9B—C8B—C7B	119.5 (3)	C12A—N1A—C13A	123.9 (3)
C9B—C8B—H8B	120.2	C12A—N1A—H1A	121 (3)
C7B—C8B—H8B	120.2	C13A—N1A—H1A	115 (3)
C7A—C8A—C9A	119.8 (3)	C12B—N1B—C13B	123.5 (3)
C7A—C8A—H8A	120.1	C12B—N1B—H1B	121 (3)
C9A—C8A—H8A	120.1	C13B—N1B—H1B	115 (3)
C6A—C7A—C8A	119.9 (3)	C18B—N2B—C14B	117.9 (5)
C6A—C7A—H7A	120.1	C14A—N2A—C18A	117.2 (4)
C8A—C7A—H7A	120.1	C5B—O1B—C4B	117.9 (3)
C6B—C7B—C8B	120.4 (3)	C5A—O1A—C4A	119.1 (3)
C6B—C7B—H7B	119.8	C11A—O2A—C6A	115.0 (3)
C8B—C7B—H7B	119.8	C11B—O2B—C6B	116.0 (3)
			
C4B—C3B—C1B—C2B	−109.8 (5)	C10B—C9B—C12B—N1B	160.6 (3)
C4A—C3A—C1A—C2A	110.6 (7)	N1A—C13A—C14A—N2A	154.4 (4)
C4A—C3A—C2A—C1A	−110.6 (7)	N1A—C13A—C14A—C15A	−26.1 (5)
C4B—C3B—C2B—C1B	107.3 (5)	N1B—C13B—C14B—N2B	−82.7 (4)
C2B—C3B—C4B—O1B	84.0 (5)	N1B—C13B—C14B—C15B	98.2 (5)
C1B—C3B—C4B—O1B	153.0 (4)	N2A—C14A—C15A—C16A	0.1 (6)
C2A—C3A—C4A—O1A	−81.6 (7)	C13A—C14A—C15A—C16A	−179.4 (4)
C1A—C3A—C4A—O1A	−153.9 (5)	N2B—C14B—C15B—C16B	2.7 (10)
O1A—C5A—C10A—C9A	−179.3 (3)	C13B—C14B—C15B—C16B	−178.3 (6)
C6A—C5A—C10A—C9A	1.3 (5)	C14A—C15A—C16A—C17A	−0.5 (8)
O1B—C5B—C10B—C9B	179.4 (3)	C14B—C15B—C16B—C17B	−0.1 (12)
C6B—C5B—C10B—C9B	−1.4 (5)	C15A—C16A—C17A—C18A	0.4 (9)
C5A—C10A—C9A—C8A	−0.6 (5)	C15B—C16B—C17B—C18B	−3.0 (13)
C5A—C10A—C9A—C12A	−178.7 (3)	C16A—C17A—C18A—N2A	0.1 (10)
C5B—C10B—C9B—C8B	0.9 (5)	C16B—C17B—C18B—N2B	3.9 (12)
C5B—C10B—C9B—C12B	178.2 (3)	O3A—C12A—N1A—C13A	2.1 (6)
C10B—C9B—C8B—C7B	−0.1 (5)	C9A—C12A—N1A—C13A	−176.3 (3)
C12B—C9B—C8B—C7B	−177.3 (3)	C14A—C13A—N1A—C12A	117.2 (4)
C10A—C9A—C8A—C7A	−0.1 (5)	O3B—C12B—N1B—C13B	−3.8 (6)
C12A—C9A—C8A—C7A	177.9 (3)	C9B—C12B—N1B—C13B	175.2 (3)
C9A—C8A—C7A—C6A	0.0 (5)	C14B—C13B—N1B—C12B	−101.4 (4)
C9B—C8B—C7B—C6B	0.0 (5)	C17B—C18B—N2B—C14B	−1.5 (11)
C8A—C7A—C6A—O2A	177.8 (3)	C15B—C14B—N2B—C18B	−1.9 (8)
C8A—C7A—C6A—C5A	0.8 (5)	C13B—C14B—N2B—C18B	179.0 (5)
O1A—C5A—C6A—C7A	179.1 (3)	C15A—C14A—N2A—C18A	0.3 (7)
C10A—C5A—C6A—C7A	−1.5 (5)	C13A—C14A—N2A—C18A	179.8 (5)
O1A—C5A—C6A—O2A	2.1 (5)	C17A—C18A—N2A—C14A	−0.4 (9)
C10A—C5A—C6A—O2A	−178.4 (3)	C10B—C5B—O1B—C4B	2.7 (5)
C8B—C7B—C6B—C5B	−0.5 (5)	C6B—C5B—O1B—C4B	−176.5 (3)
C8B—C7B—C6B—O2B	−177.4 (3)	C3B—C4B—O1B—C5B	177.4 (3)
O1B—C5B—C6B—C7B	−179.5 (3)	C10A—C5A—O1A—C4A	−2.0 (6)
C10B—C5B—C6B—C7B	1.2 (5)	C6A—C5A—O1A—C4A	177.5 (3)
O1B—C5B—C6B—O2B	−2.7 (5)	C3A—C4A—O1A—C5A	−178.0 (4)
C10B—C5B—C6B—O2B	178.1 (3)	F1A—C11A—O2A—C6A	−179.5 (3)
C10A—C9A—C12A—O3A	21.9 (5)	F2A—C11A—O2A—C6A	−63.7 (4)
C8A—C9A—C12A—O3A	−156.1 (3)	C7A—C6A—O2A—C11A	110.5 (4)
C10A—C9A—C12A—N1A	−159.6 (3)	C5A—C6A—O2A—C11A	−72.5 (4)
C8A—C9A—C12A—N1A	22.4 (5)	F1B—C11B—O2B—C6B	173.3 (3)
C8B—C9B—C12B—O3B	156.8 (3)	F2B—C11B—O2B—C6B	58.0 (4)
C10B—C9B—C12B—O3B	−20.3 (5)	C7B—C6B—O2B—C11B	−111.5 (4)
C8B—C9B—C12B—N1B	−22.2 (5)	C5B—C6B—O2B—C11B	71.5 (4)

### Hydrogen-bond geometry (Å, º)

*Cg*2, *Cg*3 and *Cg*6 are the centroids of the N2*A*/C14*A*–C18*A*, C5*A*–C10*A* and C5*B*–C10*B* rings, respectively.

**Table:**

*D*—H···*A*	*D*—H	H···*A*	*D*···*A*	*D*—H···*A*
C11*A*—H11*A*···O3*B*	0.98	2.44	3.136 (5)	128
C11*B*—H11*B*···O3*A*	0.98	2.47	3.210 (5)	132
C4*B*—H4*B*1···*Cg*3	0.97	2.87	3.689 (4)	143
C4*A*—H4*A*2···*Cg*6	0.97	2.90	3.717 (5)	143
N1*A*—H1*A*···O3*B*^i^	0.84 (4)	2.08 (4)	2.895 (4)	163 (4)
N1*B*—H1*B*···O3*A*^ii^	0.75 (4)	2.21 (4)	2.939 (4)	164 (4)
C13*A*—H13*C*···N2*B*^iii^	0.97	2.55	3.347 (5)	140
C13*A*—H13*D*···F1*A*^i^	0.97	2.52	3.294 (6)	136
C13*B*—H13*B*···*Cg*2^iv^	0.97	2.73	3.748 (4)	137

Symmetry codes: (i) *x*−1/2, −*y*+3/2, −*z*+1; (ii) *x*+1/2, −*y*+3/2, −*z*; (iii) *x*−1/2, −*y*+3/2, −*z*; (iv) *x*+1/2, −*y*+3/2, −*z*+1.
